# Effect of audio educational sessions versus self-instructional module on knowledge, practice and behavior for visually impaired adolescent females regarding menstruation

**DOI:** 10.1186/s12889-025-25868-2

**Published:** 2025-12-18

**Authors:** Maha Ramadan Ali, Fatma Zaki Mohamed Farhat, Esraa Mostafa Abd El-Aty Ibrahim

**Affiliations:** 1 Assistant professor of Maternity, Gynecology and Obstetrics nursing, Faculty of Nursing, Port−Said University, Port−Said, Egypt; 2 Assistant professor of Maternal Health Nursing and new born, Faculty of Nursing, Damietta University, Damietta, Egypt

**Keywords:** Adolescent visually impaired, Audio educational sessions, Behavior, Hygienic practices, Knowledge, Menstruation, Self instructional module

## Abstract

**Background:**

Visually impaired adolescent females (either completely blind or with partial vision loss) are a vulnerable group who require special attention and care, particularly when they begin menstruation during adolescence.

**Aim:**

Evaluate the effect of audio educational sessions versus self-instructional module on knowledge, practice and behavior for visually impaired adolescent females regarding menstruation.

**Method:**

A randomized controlled trial design was conducted at El-Nor Schools in Port-Said City. Sixty visually impaired adolescent girls who had begun menstruating were randomly assigned to two groups using purposive sampling. Data were collected using a Structured Interview Questionnaire, a Health Practices during Menstruation scale, and a Females’ Behaviors and Restrictions during Menstruation scale.

**Results:**

The SIM group’s mean score for overall knowledge of menstruation at the post-test and follow-up educational sessions was higher than that of the Audio group, with the differences being statistically significant (*P* = 0.000). In contrast, the Audio group’s mean score for overall menstrual health practices at the post-test and follow-up sessions was higher than that of the SIM group with a statistically significant difference (*P* = 0.000). Furthermore, Audio group’s mean score for overall menstruation-related behaviors at the post-test and follow-up sessions was higher than that of the SIM group with a statistically significant difference (*P* = 0.000). In addition, a positive correlation was observed between the pre- and post-test total scores of knowledge, health practices, and behaviors in both study groups, with the differences being statistically significant (*P* ≤ 0.001).

**Conclusion:**

Audio educational sessions, when combined with a Braille-based self-learning module, are both essential and highly effective in improving menstruation-related knowledge, practices, and behaviors among visually impaired adolescent girls.

**Trial registration:**

The study protocol was registered with the Research Ethics Committee of the Faculty of Nursing, Port Said University, under code number NUR13, on 7/4/2022.

## Background

Visual impairment is a condition that not only limits physical abilities but also has profound psychological and social effects. It disrupts daily routines and developmental processes in visually impaired girls, influencing their families and communities as well. Because vision is crucial for learning and interpreting experiences, its loss represents one of the most serious sensory disabilities [1]. Early intervention and rehabilitation programs are essential to help visually impaired adolescent girls improve their daily activities, education, communication, and overall lifestyle [2].

Menstruation is a normal physiological process and an important indicator of female reproductive health [3]. However, cultural and spiritual beliefs strongly influence how it is understood and managed within different societies [4]. Misconceptions and traditional taboos surrounding menstruation are widespread and often transferred across generations. While some customs have cultural value, others may negatively affect girls’ health and well-being [5].

Proper menstrual hygiene is critical for maintaining reproductive health and preventing infections. Given their physical limitations, visually impaired girls are more vulnerable to menstrual and gynecological problems; adopting correct hygiene behaviors can greatly reduce these risks [6]. Hygienic menstrual practices involve regular bathing, handwashing, changing and cleaning clothes, safe pad disposal, and managing discomfort using warm compresses, exercise, or relaxation techniques [7].

Raising awareness about menstrual hygiene is essential to promoting the health and confidence of adolescent girls worldwide [8]. For visually impaired individuals, self-instructional modules (SIMs) and the Braille system remain the most important tools for independent access to knowledge and education. Braille allows individuals to explore and understand the world through touch, making it a vital medium for reading and writing. As Wang and Takeda [9] explained, the system was developed to help visually impaired people overcome barriers and acquire the knowledge needed to participate productively in society.

In addition, audio-based education serves as an effective instructional strategy for visually impaired adolescent girls, facilitating their acquisition of knowledge relevant to health promotion. It is recognized as a powerful method of teaching and training, equipping students with essential information to enhance learning and interpersonal development [10]. Audio educational programs further benefit visually impaired adolescents by strengthening self-evaluation, peer relationships, decision-making, and self-care skills. By focusing on factual knowledge about menstruation and hygienic practices, such programs can improve understanding of puberty and menstruation, while also empowering girls to challenge myths, misconceptions, and harmful restrictions [11].

Nurses play a central role in supporting the health of visually impaired adolescent girls by providing education on appropriate menstrual hygiene practices. They contribute not only through direct knowledge transfer and supervision but also by ensuring access to school-based health services. This includes health education, screenings, referrals to specialized or home-based care, and collaboration with families, schools, and community members. In doing so, school nurses establish vital links between students and healthcare professionals, promoting a healthy school environment and addressing the unique needs of visually impaired adolescent girls [12].

### Significance of the study

According to reports from Abdelazeem et al. [13] and the World Health Organization (WHO) [14], the number of visually impaired individuals in Egypt has reached approximately 3 million. Within the framework of Egypt’s 2030 strategy, particularly the fifth axis of the social dimension, it is emphasized that major segments of society—especially visually impaired adolescent females—remain disadvantaged. Therefore, providing care and support to this group is crucial to achieving social equity for all members of the community.

Vision loss limits the ability of visually impaired adolescent girls to perform daily tasks and to learn through observation and practice of self-care activities. Thus, it is essential not only to provide supportive educational resources to enhance their knowledge and awareness of menstruation but also to assess their existing knowledge and practices [15]. Moreover, this study was undertaken because of the scarcity of Egyptian research that investigates the comparative effects of audio-educational sessions and self-instructional modules using the Braille method on the knowledge, practices, and behaviors of visually impaired adolescent girls regarding menstruation.

### Aim of the study

The aim of the present study was to evaluate the effect of audio educational sessions versus self-instructional module on knowledge, practice and behavior for visually impaired adolescent females regarding menstruation.

### Research objectives

This aim will be fulfilled through:

1. Assess the effect of audio educational sessions versus self-instructional module on knowledge related to menstruation.
2. Assess the effect of audio educational sessions versus self-instructional module on hygienic practices during menstruation.
3. Assess the effect of audio educational sessions versus self-instructional module on health behavior of visually impaired adolescent females regarding menstruation.

### Operational definitions

#### Self-Instructional Module (SIM)

The SIM provides information on knowledge, adaptive health practices, and behaviors for visually impaired adolescent females. The content covers the anatomy and physiology of the female reproductive system, the menstrual cycle, diet during menstruation, hygienic practices, and is delivered through printed Arabic booklets in Braille.

#### Audio educational sessions

These sessions consist of a CD-ROM with recorded audio materials focusing on menstrual hygiene and the management of minor discomforts during menstruation.

## Method

### Research design

This study was conducted using a randomized controlled trial design with two parallel groups: the Audio group and the Self-Instructional Module (SIM) group.

### Study setting

The research was conducted at El-Nor School in Port-Said City. The school includes three educational levels: primary, preparatory, and secondary. It offers a range of benefits to its students, such as free education, free Braille books, free school uniforms and clothing, daily meals (including pasta with chicken or meat), and dormitory accommodation for students from outside the city, all under the supervision of the school staff.

### Study sample

Regardless of the cause of their disabilities, a purposive sample of sixty visually impaired menstruating adolescent females, affiliated with Port Said City, enrolled in the aforementioned school, and meeting the inclusion criteria, was included in the study. The participants were equally allocated into two groups through simple randomization using random number tables to ensure an equal chance of assignment:

- Group A (Self-Instructional Module group): received the Braille version of the module.
- Group B (Audio group): received scheduled lessons via audio CD-ROM.

### Inclusion criteria

The following criteria were used to include visually impaired adolescent girls from the aforementioned school in the study:

- Adolescents aged 12–19 years.
- Those who had attained menarche.
- Unmarried adolescent females.
- Adolescents with the ability to communicate effectively.

### Exclusion criteria

- Adolescents with visual impairment who also had verbal or hearing impairments.
- Visually impaired adolescents with mental disabilities.

### Sample technique

According to a comprehensive assessment conducted by the Directorate of Special Education in Port-Said, only one school for visually impaired girls was identified in the city. A review of the registration files of students across three preparatory and secondary grade levels at this school revealed that a total of sixty visually impaired girls were enrolled at Al-Nor School in Port-Said City.

### Tools of data collection

Three tools were used to collect data at this study:

#### Tool I: structured interview

The tool was originally designed by Heiba, AbdElmenim, and Mohammed [16] and was later modified by the researcher. It consisted of multiple-choice questions written in the Arabic language, developed based on a review of relevant literature and expert opinions. The tool was divided into three main sections that assessed the following:

#### Section one

This section consisted of twelve multiple-choice questions addressing the general characteristics of the visually impaired females included in the study. It assessed the socioeconomic status (SES) of the participants across seven domains: the girl’s education, parental education and occupation, family size, household possessions, household sanitation, and family income.

#### Section two

This section focused on the menstrual cycle characteristics and health complaints reported by the participants. It included multiple-choice questions on the following aspects: age at menarche, response to menarche, menstrual regularity, duration of menstrual flow, and intermenstrual interval. Additionally, two questions were included to evaluate menstrual complaints such as premenstrual syndrome and other menstrual symptoms. Furthermore, two more questions were designed to assess current health complaints, with a focus on the features of vaginal discharge, including odor, color, consistency, texture, and any accompanying signs of infection.

#### Section three

This section was divided into two parts:

##### Part 1

Assessed the knowledge of visually impaired adolescent girls about menstruation. It comprised fifteen multiple-choice questions and four open-ended questions addressing the following topics: anatomy of the female reproductive system; physical changes during puberty; age at menarche; definition of menstruation; source of menstrual blood; normal menstrual interval and duration; signs and symptoms associated with menstruation; complications of neglecting menstrual hygiene; and dietary practices, including foods and herbs to consume or avoid during menstruation.

*The menstrual knowledge of the females was evaluated using the following scoring system:*

The interview questionnaire items formed the basis of the scoring system. The researcher used a model answer key, developed from relevant literature, to evaluate the participants’ responses. Each question was scored from 0 to 1 point, with one point awarded for a correct answer and zero for an incorrect or “don’t know” response. For open-ended questions, participants were free to respond and elaborate; their answers were assessed by the researcher, who assigned scores according to accuracy.

The overall knowledge score was calculated by summing all correct responses (57 points, representing 100% of the total). Scores were then classified into three categories:

- *Poor*: Less than 50% of the total score (<28.5 marks).
- *Fair:* 50% to <75% of the total score (28.5 to <42.75 marks).
- *Good:* ≥75% of the total score (42.75–57 marks).

##### Part 2

This section consisted of four questions exploring the sources from which girls obtained information about menstruation, the timing of when they first received this information, and the challenges they faced in seeking medical care for menstrual problems.

#### Tool (II): health practices during menstruation assessment sheet

It was adapted from Heiba et al. [16] and modified by the researcher. The instrument, written in Arabic, was used to assess the menstrual health practices of adolescent girls.

*There were two sections to it:*

- *The first section* addressed the hygienic practices of visually impaired adolescent females during menstruation. It included fifteen multiple-choice questions covering topics such as personal cleaning techniques, direction of perineal care, maintaining dryness of the perineal area, bathing during menstruation, types of sanitary towels used and their frequency of change, the use of talcum powder and perfumed substances on the perineum, pubic hair removal, handwashing, types of underwear and their frequency of change, methods of cleaning, and techniques for disposing of sanitary pads.
- *The second section* focused on healthy nutrition during menstruation, comprising eleven multiple-choice questions related to the types of food and fluids that should be consumed or avoided during menstruation.

##### Menstrual hygiene practices among the students were assessed using the following scoring system

All responses were coded, with one point awarded for each healthy practice and zero for each unhealthy practice. The total possible score for practicing both healthy diet and menstrual hygiene during menstruation was 26 points (100%).

The final practice scores were categorized as follows:

- *Healthy (good) practice*: ≥ 75% of the total score (≥ 19.5 points).
- *Unhealthy (poor) practice*: < 75% of the total score (< 19.5 points).

##### Tool (III): females behaviors and restrictions during menstruation assessment sheet

It was adapted from Heiba et al. [16], with modifications made by the researcher. The instrument, written in Arabic, consisted of eight questions designed to evaluate the menstrual habits and restrictions of the participating females. These included attending school, performing household tasks, visiting holy sites, interacting with family members and friends during menstruation, engaging in physical activities, taking painkillers or herbal remedies, and seeking advice for amenorrhea.

*The scoring system for menstrual behaviors was as follows:*

Each response was coded, with one point assigned for every positive behavior and zero for every negative behavior. The total possible score for menstrual behaviors was 8 points (100%).

- *Good behavior*: ≥ 75% (≥ 6 points)
- *Poor behavior*: < 75% (< 6 points). This classification represented the final behavior score.

#### Tool content validity

The researcher developed the data collection tools, which were subsequently reviewed by a panel of five experts from the nursing and medical faculties at Port Said and Suez Canal Universities, specializing in obstetrics, gynecology, and maternity. The aim of this review was to evaluate the tools’ appropriateness, comprehensiveness, relevance, and clarity. Experts were also asked to provide feedback on the format, layout, and coherence of the instruments. Based on their recommendations, the item related to “color of vaginal discharge and redness of the labia” was removed, and the necessary modifications were made accordingly.

#### Content reliability

Cronbach’s alpha coefficient was calculated to assess reliability. The results demonstrated high reliability, with values of 0.76 for knowledge, 0.89 for practice, and 0.80 for behavior.

#### Ethical approval

According to code NUR 13 on 7/4/2022, the Research Ethics Committee of the Faculty of Nursing, Port Said University, formally reviewed and approved the study. In addition, approval was obtained from the head manager of the study setting prior to data collection. All participants and their parents received a clear explanation from the researchers regarding the purpose of the study. Each adolescent girl was informed of her right to freely choose whether or not to participate. Oral consent was obtained from the parents of all visually impaired adolescent girls via telephone. In addition, each female participant provided informed consent to participate. They were assured that all data would remain confidential and be used solely to achieve the objectives of the study. Participants’ privacy was strictly maintained at all times.

##### Administrative design

Formal administrative approval was obtained from the Dean of the Faculty of Nursing prior to conducting the study in the previously mentioned setting in Port Said Governorate. Official permission was also obtained from the directors of the setting to facilitate data collection and coordination. The administrative bodies were informed about the study objectives and implementation plan to ensure smooth cooperation during the research process. They were also assured that all collected information would remain confidential and be used solely for research purposes.

#### Pilot pretest

Six randomly selected female participants from the aforementioned school, representing 10% of the study population, were included in the pilot pretest. This pilot testing was conducted to assess the applicability, clarity, and relevance of the developed tool, as well as to estimate the time required to complete the questionnaire. Based on the findings, the questionnaire was revised, with certain items added and others removed, particularly in the sections related to students’ knowledge and practices. Consequently, the participants in the pilot pretest were excluded from the main study sample.

### Field work

The preparatory, assessment, instructional session planning, implementation, and evaluation phases constituted the five stages of the study. Data were collected over a six-month period, from October 2022 to March 31, 2023.

#### The preparatory stage

A group of experts in maternity nursing and obstetric medicine confirmed the content validity of the developed tool, the educational sessions, and the material. Following this, the tools were established, the instructional sessions using audio CD-ROM and the self-instructional module (an Arabic booklet produced by the Braille method) were developed, and finally, a pilot pretest was carried out to assess the applicability of the study tools.

#### Phase of assessment (pre-test phase)

The researcher carried out this phase from the beginning of October 2022 to the end of the month. Once the instruments were ready, the study sample was selected based on the predetermined standards. After introducing herself, the researcher clearly presented the study objectives to the participants in a simple and easily understandable manner. Following their consent to participate, she conducted individual interviews with the visually impaired adolescent females. She then asked them to complete a questionnaire form that she had provided. Complete confidentiality of all gathered data was rigorously guaranteed.

The next step was collecting baseline data. The study groups were given a pre-tested questionnaire to assess their current menstrual practices, knowledge, and behaviors. Data collection was conducted during the school day in the students’ free time. Schools were visited three days a week, from 9 a.m. to 1 p.m.

#### Phase of planning

Based on the data from the first assessment and the literature review, the researcher began creating organized audio educational sessions for the audio group. Under supervisors’ guidance, the researcher also created structured audio CD-ROM instructional sessions.

After reviewing the literature, the researcher developed three organized audio-educational sessions that were presented over three consecutive days. They were written in simple Arabic to help visually impaired adolescent girls gain more knowledge about the anatomy and physiology of the female reproductive organs, the menstrual cycle, healthy nutrition related to menstruation, and personal hygiene during the menstrual cycle.

Furthermore, the researcher created an Arabic booklet using the Braille method for the self-instructional module. The booklet’s content was written in easily understandable Arabic, and permission to print the educational booklet in Braille was obtained from the director of the Al-Nor Association for the Blind as standard practice. The booklet covers information about the anatomy and physiology of the reproductive system, the menstrual cycle, healthy nutrition related to menstruation, and personal hygiene during the menstrual cycle.

Since the Al-Nor Association for the Blind has a printer capable of producing educational materials in Braille, the researcher provided a soft copy of the instructional booklet to the organization. The material was then printed on Braille paper and compiled into a booklet using the Braille technique.

#### Phase of implementation

**Fig. 1:**
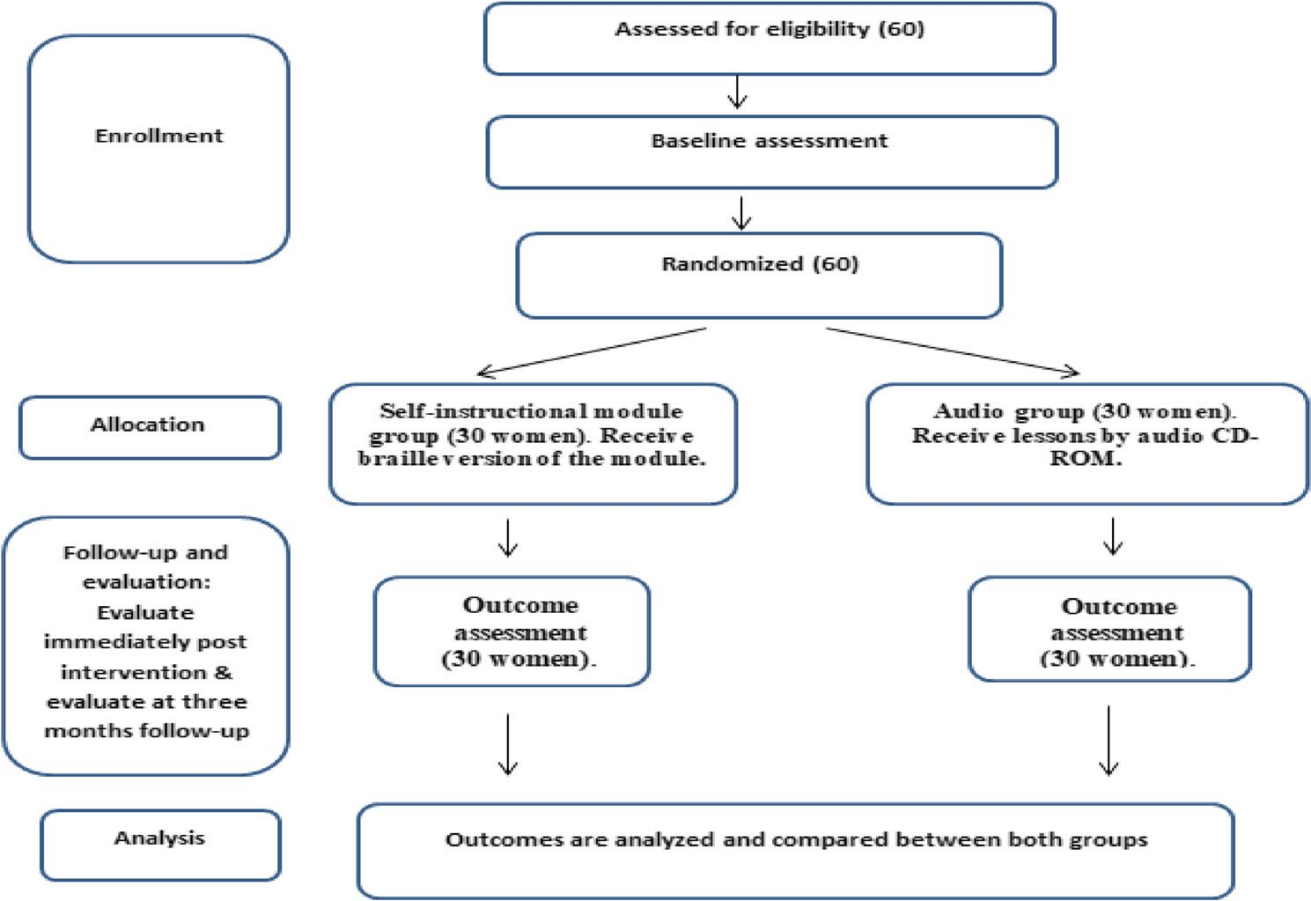
CONSORT diagram illustrating the random allocation process and participation of visually impaired adolescent girls throughout the study
- Eight subgroups, four for each of the audio and SIM groups, were created, with 7–8 students per subgroup based on the total number of participants.
- For the audio group, the structured audio CD-ROM training sessions developed for this study were conducted in computer labs and classrooms at the designated schools in Port-Said. Each member of the SIM group received a self-instructional module created using the Braille method and written in simple Arabic.
- The researcher relied on coordinating with the school director to obtain information regarding the schedules and times of classes of female students in order to conduct interviews. Additionally, to allow the researcher to interview female students, cooperation was established with the teachers.
- The researcher presented herself to the visually impaired adolescent females on the recruitment day, when the baseline assessments for the two groups were conducted, and she explained to them the purpose of the educational sessions.
- After administering a standardized questionnaire, the researchers evaluated the sociodemographic information of each participant, their menstrual characteristics, their knowledge and practice of menstrual hygiene, and any mild discomfort associated with it in the two groups.
- The researchers took around fifteen minutes to perform the baseline assessment for each participant, followed by a thirty-minute break.
- The first instructional session in the audio group began with an audio lecture delivered by the researchers in understandable Arabic for the participants.
- The primary aim of the initial educational session was to impart fundamental scientific knowledge regarding the anatomy and physiology of the reproductive system and the menstrual cycle. On the second day of enrollment, a quick recap of the first session was presented, followed by the second educational session, which focused on highlighting the significance of menstrual hygiene products.
- On the third enrollment day, a quick recap of the second session was presented, followed by the third instructional session, which aimed to show how to handle moderate menstrual discomfort with easy-to-implement solutions.
- At the end of every audio group session, all questions from the participants were addressed by the researchers through group discussions. A pause of approximately 45 min was observed between each educational session.
- The SIM groups read a portion of an instructional booklet in Braille, which was created in simple Arabic and distributed to all participants. Each subgroup was given a week to read the booklet. Additionally, after reading the booklet, the researchers conducted group discussions to address all questions from the subgroup members.
- The implementation of the instructional sessions lasted for two months, according to the educational content, specifically from November to the end of December 2022, for each of the two groups.

- First, a letter of authorization was obtained from Al-Nor Schools for the Blind to conduct the study, and consent was obtained from the adolescent girls who agreed to participate.
- The participants were divided into two groups (SIM and audio groups) using a random number table; however, blinding of participants was not feasible due to the nature of the educational intervention (Fig. 1).

#### Phase of evaluation

Following the reading of the SIM group’s booklet and the conclusion of the audio group’s third and final audio educational session, the participants were reassessed for their knowledge of menstrual hygiene and management of minor complaints (Immediate post-test for knowledge). The participants were reassessed for their practices related to menstrual hygiene and management of minor discomfort one month after enrollment (Post-test one for practice). The participants’ knowledge and practices regarding menstruation were then reassessed by the researcher three months following their initial enrollment (Post-test two for knowledge and practice). Approximately thirty minutes were required for each visually impaired female to complete the assessment.

The assessment phase lasted for three months, from the end of December 2022 to the end of March 2023, as originally planned. The trial was not stopped early, and no changes were made to its methods or objectives during this period.

The educational interventions were well tolerated, and no significant harms or unintended effects were observed in either the Braille or the audio group throughout the study period.

### Statistical analysis

The data were entered into the computer, and analysis was performed using IBM SPSS software package, version 20.0 (IBM Corp., Armonk, NY). Numbers and percentages were used to describe the qualitative data. The normality of the distribution was assessed using the Shapiro-Wilk test. Quantitative data were presented as range (minimum and maximum), mean, standard deviation, and median with interquartile range (IQR). Results were considered statistically significant at the 5% level.

The following tests were used to determine the significance of differences: the chi-square and Student’s t-test to compare two groups for normally distributed quantitative variables, the F-test (ANOVA) to compare more than two categories for normally distributed quantitative variables, repeated measures ANOVA to compare more than two time points for normally distributed quantitative variables, and the Pearson correlation coefficient to assess the relationship between two normally distributed quantitative variables.

## Results

Table 1 illustrates the distribution of visually impaired adolescent females according to their general characteristics. The mean age was 14.36 ± 2.14 in the SIM group compared to 13.98 ± 1.95 in the Audio group, with most participants in both groups being at the preparatory stage. Regarding parental education, more than half of the fathers in the SIM group (58.0%) had a university degree or higher compared to 58.9% in the Audio group. Meanwhile, more than one-third of the mothers in the SIM group (42.0%) had secondary education compared to 51.7% in the Audio group. Regarding parental occupation, the majority of fathers (91.6%) in the SIM group were employed compared to 90% in the Audio group, while most mothers (85.0%) in the SIM group were housewives compared to 77.6% in the Audio group.

**Table 1:** Distribution of the general characteristics of the visually impaired adolescent females among the studied groups (*n* = 60)

General characteristics	SIM Group (30)		Audio Group (30)	
	No.	%	No.	%
Age				
12–14 year	36	60.0	37	62.3
15–17 year	19	32.0	16	26.4
18–20 year	5	8.0	7	11.3
Mean ± SD	14.36 ± 2.14		13.98 ± 1.95	
Current educational stage				
Preparatory stage	49	82.0	37	61.4
Secondary stage	11	18.0	23	38.6
Education and culture of parents				
Father				
Not read and write	8	14.0	3	4.2
Read and write	7	12.0	5	7.8
Basic education	0	0.0	0	0.0
Secondary	10	16.0	17	29.1
University and above	35	58.0	35	58.9
Mother				
Not read and write	7	12.0	1	1.6
Read and write	18	30.0	5	8.3
Basic education	0	0.0	0	0.0
Secondary	10	16.0	23	38.4
University and above	25	42.0	31	51.7
Occupation				
Father				
Not work	5	8.4	6	10.0
Work	55	91.6	54	90.0
Mother				
House wife	51	85.0	47	77.6
Work	9	15.0	13	22.4
Place of residence				
Rural areas	16	26.0	20	33.8
Urban areas	44	74.0	40	66.2
Number of family members				
4–5	35	58.0	31	50.9
6–8	18	30.0	24	40.1
>8	7	12.0	5	9.0
Arrangement among siblings				
First	18	30.0	16	26.0
Second	27	45.0	23	39.0
Third	8	13.0	15	25.0
Fourth	7	12.0	6	10.0
Number workers family members				
One person	46	76.0	55	91.6
Two persons	14	24.0	5	8.4
Family income				
Enough	32	54.0	21	35.8
Not enough	28	46.0	39	64.2
Number of rooms				
Two	16	26.0	19	31.0
Three	44	74.0	41	69.0

Continuing, Table 1 shows that nearly three-quarters of the visually impaired adolescent females (74.0%) in the SIM group resided in urban areas compared to 66.2% in the Audio group. More than half of the SIM group (58%) had four to five family members compared to 50.9% in the Audio group. Over one-third of the participants were second-born among their siblings in the SIM and Audio groups (45.0% and 39.0%, respectively). Regarding economic status, more than half of the SIM group (54.0%) reported sufficient economic status, while 64.2% of the Audio group reported insufficient economic status. Additionally, nearly three-quarters of the SIM group (74.0%) had three rooms in their household compared to 69% in the Audio group.

Table 2 reveals that the mean age at menarche was 12.50 ± 1.46 in the SIM group compared to 12.9 ± 1.54 in the Audio group. Regarding reactions to menarche, most females (80.0%) in the SIM group felt fear during their first menstruation compared to 85.0% in the Audio group. Additionally, more than three-quarters of females in the SIM group (74.0%) had irregular menstruation compared to 71.7% in the Audio group. Concerning the duration of menstruation, more than two-thirds of females in both groups (68.0%) had periods lasting 3 to 7 days.

**Table 2:** Distribution of the visually impaired adolescent females according to their menstruation characteristics among the studied groups (*n* = 60)

Menstruation characteristics	SIM Group (30)		Audio Group (30)	
	No.	%	No.	%
Age of menarche (in years)				
< 10–11 year	16	26.0	15	25.0
12 year	16	26.0	17	28.0
13–15 year	28	48.0	28	47.0
More than 15 year	0	0.0	0	0.0
Mean ± SD.	12.50 ± 1.46		12.9 ± 1.54	
Reaction at menarche				
Feeling fear	48	80.0	51	85.0
Feeling happiness	2	4.0	2	3.0
Feeling ashmed	10	16.0	7	12.0
Regularity of the menstruation (that is, it comes every month)				
Yes	16	26.0	17	28.3
No	44	74.0	43	71.7
Frequency of menstruation				
Less than 21 day	0	0.0	0	0.0
From 21:35 day	44	74.0	41	69.0
More than 35 day	0	0.0	0	0.0
Irregular interval	16	26.0	19	31.0
Mean ± SD.	27.54 ± 4.38		27.42 ± 4.25	
Duration of menstruation				
Less than 3 days	7	12.0	3	5.0
From 3–7 days	41	68.0	41	68.0
More than 7 days	12	20.0	16	27.0
Mean ± SD.	5.18 ± 1.32		5.14 ± 1.21	

As shown in Table 3, about three-quarters of females in the SIM group (73.3%) reported experiencing premenstrual symptoms compared to 70.0% in the Audio group. More than three-quarters of these females (77.2%) in the SIM group reported abdominal cramps compared to 85.7% in the Audio group, which was considered the most common complaint. In addition, about three-quarters of females in the SIM group (73.3%) reported complaints during menstruation compared to 70.0% in the Audio group. All participants in both groups (100.0%) reported abdominal pain. Moreover, most females in the SIM group (81.8%) reported back pain compared to 80.9% in the Audio group.

**Table 3:** Distribution of the visually impaired adolescent females according to self-reported complaints of menstruation among the studied groups (*n* = 60)

Self-Reported complaints of menstruation	SIM Group (30)		Audio Group (30)	
	No.	%	No.	%
Associated Premenstrual symptoms				
No	8	26.7	9	30.0
Yes	22	73.3	21	70.0
Symptoms	(*n* = 22)		(*n* = 21)	
Excess worry	4	18.1	2	9.5
Fatigue and tiredness	14	63.6	11	52.3
Breast tenderness	7	31.8	6	28.5
Abdominal cramps	17	77.2	18	85.7
The appearance of pimples on the face	9	40.9	10	47.6
Headache	1	4.5	1	4.7
Nervousness	10	45.4	10	47.6
Complains during menstruation				
No	8	26.7	9	30.0
Yes	22	73.3	21	70.0
Type of complain	(*n* = 22)		(*n* = 21)	
Feeling tired	9	40.9	6	28.5
Back pain	18	81.8	17	80.9
Anorexia	9	40.9	5	23.8
Abdominal pain	22	100.0	21	100.0

**Fig. 2:**
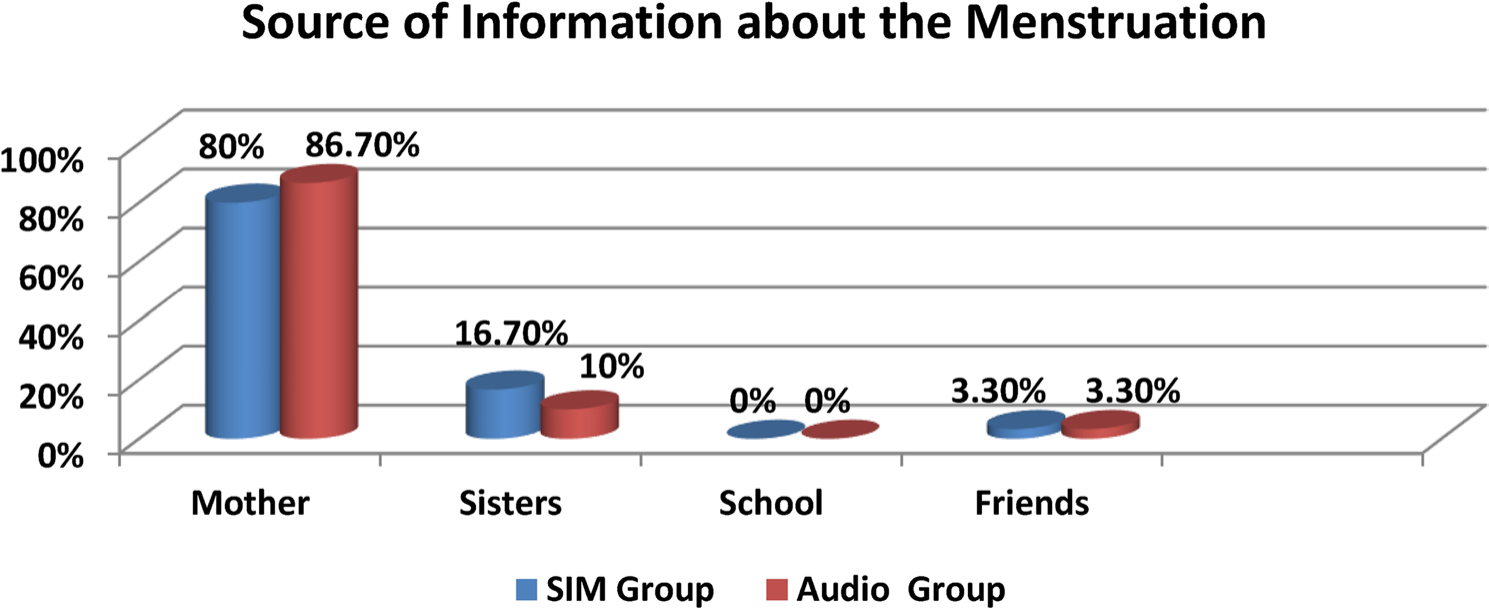
Distribution of the visually impaired adolescent females according to source of information about menstruation among the studied groups (*n* = 60)

As illustrated in Fig. 2, most visually impaired adolescent females (80.0%) in the SIM group reported that their mothers were their source of information regarding menstruation, compared to 86.7% in the Audio group.

Table 4 shows that the mean total knowledge score regarding menstruation in the SIM group improved at post-intervention and follow-up (1.95 ± 0.097 and 1.94 ± 0.180, respectively) compared to the pretest (0.44 ± 1.491). Similarly, the mean total knowledge score in the Audio group increased at post-intervention and follow-up (1.49 ± 0.712 and 1.39 ± 0.773, respectively) compared to the pretest (0.35 ± 0.475). Furthermore, mean total knowledge scores were higher in the SIM group than in the Audio group, with the differences being statistically significant (*P* = 0.000).

**Table 4:** Knowledge mean scores of the visually impaired adolescents females regarding menstruation throughout the program phases among the studied groups (*n* = 60)

Knowledge regarding menstruation	SIM Group (30)			Audio Group (30)			F	*p*-value
	Pre	Post immediate	Post after 3 month	Pre	Post immediate	Post after 3 month		
	Mean ± SD.	Mean ± SD.	Mean ± SD.	Mean ± SD.	Mean ± SD.	Mean ± SD.		
Biological health aspects of menstruation	0.20 ± 4.03	2.0 ± 0.000	1.97 ± 0.181	0.23 ± 0.427	1.42 ± 0.766	1.35 ± 0.799	63.250	0.000*
Menstruation	0.43 ± 0.500	1.82 ± 0.390	1.85 ± 0.360	0.42 ± 0.497	1.37 ± 0.736	1.25 ± 0.751	194.792	0.000*
Complaints and complications of neglecting menstrual hygiene	0.25 ± 0.437	2.0 ± 0.000	1.97 ± 0.181	0.37 ± 0.486	1.68 ± 0.624	1.60 ± 0.718	163.036	0.000*
Proper nutrition during menstruation	0.89 ± 0.999	2.0 ± 0.000	2.0 ± 0.000	0.38 ± 0.490	1.50 ± 0.725	1.38 ± 0.825	194.792	0.000*
Overall Knowledge regarding menstruation	0.44 ± 1.491	1.95 ± 0.097	1.94 ± 0.180	0.35 ± 0.475	1.49 ± 0.712	1.39 ± 0.773	153.967	0.000*

*Statistically significant at *p* ≤ 0.05 F: F test (ANOVA) with repeated measures

Table 5 shows that the mean total health practice score regarding menstruation in the SIM group improved at post-intervention and follow-up (0.67 ± 0.471 and 0.72 ± 0.449, respectively) compared to the pretest (0.11 ± 0.213). Similarly, the mean total health practice score in the Audio group increased at post-intervention and follow-up (0.90 ± 0.290 and 0.965 ± 0.090, respectively) compared to the pretest (0.51 ± 0.375). Moreover, the mean total health practice scores regarding menstruation were higher in the Audio group than in the SIM group, with the differences being statistically significant (*P* = 0.000).

**Table 5:** Health practice mean score of the visually impaired adolescent females during menstruation pre-post and follow up program among the studied groups (*n* = 60)

Health practices during menstruation	SIM Group (30)			Audio Group (30)			F	*p*-value
	Pre	Post after 1 month	Follow up after 3 months	Pre	Post after 1 month	Follow up after 3 months		
	Mean ± SD.	Mean ± SD.	Mean ± SD.	Mean ± SD.	Mean ± SD.	Mean ± SD.		
Hygienic practice	.23±.427	.65±.481	.70±.462	.18±.390	.85±.360	.97±.181	1.780	0.000*
Nutritional practice	.00±.000	.70±.462	.75±.437	.85±.360	.95±.220	.96±.00	1.780	0.000*
Overall Health practices during menstruation	.11±.213	.67±.471	.72±.449	.51±.375	.90±.290	.965±.090	1.780	0.000*

F: F test (ANOVA) with repeated measures

p: *p* value for comparing between the studied periods

*: Statistically significant at *p* ≤ 0.05

Table 6 shows that the mean total menstrual behavior score in the SIM group improved at post-intervention and follow-up (0.60 ± 0.494) compared to the pretest (0.11 ± 0.323). Similarly, the mean score in the Audio group increased at post-intervention and follow-up (0.66 ± 0.475) compared to the pretest (0.08 ± 0.278). Moreover, the mean total menstrual behavior scores were higher in the Audio group than in the SIM group, with the differences being statistically significant (*P* = 0.000).

**Table 6:** Behaviors mean score of the visually impaired adolescent females during menstruation pre-post and follow up program among the studied groups (*n* = 60)

Behaviors	SIM Group (30)			Audio Group (30)			F	*P*
	Pre	Post after 1 month	Follow up after 3 months	Pre	Post after 1 month	Follow up after 3 months		
	Mean ± SD.	Mean ± SD.	Mean ± SD.	Mean ± SD.	Mean ± SD.	Mean ± SD.		
Menstrual behaviors and restrictions	.11±.323	.60±.494	.60±.494	.08±.278	.66±.475	.66±.475	F=1.278	0.000*

F: F test (ANOVA) with repeated measures

*: Statistically significant at *p* ≤ 0.05

Table 7 demonstrates the correlations between the studied females’ total scores of knowledge, health practices, behaviors, and restrictions at both pre- and post-test. Positive correlations were observed, with the differences being statistically significant (*P* ≤ 0.001).

**Table 7:** Correlation between overall score level of knowledge regarding menstruation, health practices, behaviors and restrictions during menstruation among the studied groups at pre and post test (*n* = 60)

Variables		Knowledge regarding menstruation in SIM group		Knowledge regarding menstruation in Audio group	
		Pre	Post after 3 month	Pre	Post after 3 month
Health practices during menstruation in SIM group	R	.546	.948	----	----
	P	0.000^*^	0.000^*^	----	----
Behaviors and restrictions during menstruation in SIM group	R	.264	.618	----	----
	P	0.001^*^	0.000^*^	----	----
Health practices during menstruation in Audio group	R	----	----	.466	.838
	P	----	----	0.000^*^	0.000^*^
Behaviors and restrictions during menstruation in Audio group	R	----	----	.445	.690
	P	----	----	0.000^*^	0.000^*^

r: Pearson coefficient

*: Statistically significant at *p* ≤ 0.05

## Discussion

A girl’s menstruation may mark the transition from childhood to womanhood. It is a very delicate turning point in a woman’s life, and even typically developing adolescent girls may not know how to manage their periods properly [17]. Due to vision loss, visually impaired teenage girls are less able to perform daily tasks and have limited opportunities to learn through observation and participation in self-care activities [18]. Therefore, the aim of this study was to assess the effect of audio educational sessions versus a self-instructional module on the knowledge, practices, and behaviors of visually impaired adolescent females regarding menstruation.

According to the study’s findings, almost half of the female participants in both the SIM and Audio groups experienced menarche between the ages of 13 and 15. The majority in both groups felt fear during their first menstruation as a reaction to menarche. Over two-thirds of the girls in the study experienced irregular menstruation. Regarding menstrual duration, almost two-thirds of the female participants in both groups had periods lasting between three and seven days.

These findings may be explained by the sensitivity of this age group, which falls between the middle and late phases of adolescence and is associated with higher health risk behaviors. These results are consistent with the study by Kumbhar & Bhore [19], which examined the impact of an audio CD-ROM versus a self-instructional module on the knowledge and self-reported reproductive health behaviors of 12 blind adolescents enrolled in specially selected blind schools in India. The study found that 75% of the adolescents were aged 15–18, over half had menstrual cycles lasting three to five days, half experienced intervals of 28–35 days, and most had moderate menstrual blood volume.

Furthermore, Abd Elkodoos et al. [15] reported that the mean age of the participating girls was 14.8 ± 1.4 years, with over half of them aged between 15 and 18 years. These findings support the current study’s results.

The current study found that almost more than two third of the examined visually impaired adolescent females in both groups self-reported experiencing premenstrual symptoms related to their menstrual complaints. Abdominal cramps affected over two third of the participants. Furthermore, the majority reported pain, and all of them experienced abdominal pain.

Minor premenstrual syndrome and mild complaints during menstruation are common physiological symptoms associated with the menstrual cycle, which could explain these findings. These results are consistent with the study by McGregor and Unsworth [12], titled “Menstrual hygiene management strategies used by girls who are blind or have low vision,” which reported that all study participants experienced premenstrual syndrome and menstruation-related problems.

Moreover, the majority of participants in Ghazy & Fathy’s [20] study on the “Effect of an audio drama-based educational program on healthy lifestyle practices among visually impaired students” experienced menstrual irregularities.

Regarding sources of information about menstruation among the visually impaired adolescent girls in the current study, the results showed that most participants reported receiving information from their mothers.

This finding may be explained by the fact that visually impaired teenage girls often maintain close social relationships primarily with their mothers, who are considered their main trusted source of guidance, compared to friends, other family members, or teachers.

This finding is consistent with Abd Elmegaly, Attia, and Soliman [21], who reported that 25% of students considered their mother as their primary source of information regarding puberty. Similarly, Jayanthi [6] found that most visually impaired adolescent schoolgirls obtained knowledge about menstruation from their mothers, with a smaller proportion gaining information from schools and the media.

Regarding the biological health aspects of menstruation among the studied visually impaired adolescent females in relation to the pre-, post-, and follow-up educational sessions, the current study showed that while approximately half of the Audio group demonstrated good knowledge about menstruation at post- and follow-up sessions, the vast majority of the SIM group of visually impaired adolescent females exhibited good knowledge at post- and follow-up sessions. This difference was highly statistically significant (*P* = 0.000).

The researchers believe that this improvement may be attributed to the effectiveness of the educational sessions in raising the participants’ knowledge levels. Additionally, a reproductive health book was provided to each teenage girl in the school library. The book was translated into Braille, allowing the girls to read it at any time and apply the knowledge to improve their lives. However, the Audio group required extra time at the Blind School to listen to the audio CD-ROM.

These findings are supported by Kumbhar & Bhore [19], who reported that during the pretest, 75% and 91.67% of blind teenage girls in the Audio-CD ROM group and SIM group, respectively, had inadequate knowledge of reproductive health. Posttest results showed that 50% of the SIM group and 16.67% of the Audio-CD ROM group achieved good knowledge. Furthermore, the mean posttest knowledge score was 14.00 in the SIM group compared to 9.16 at pretest, with a significant difference (*p* = 0.000). Similarly, the Audio-CD ROM group’s mean knowledge score increased from 9.30 at pretest to 13.10 at posttest, also with a significant difference (*p* = 0.000).

Furthermore, the research conducted by Abd Elkodoos et al. [15] demonstrated that most teenage girls exhibited a good level of knowledge in both the immediate posttest and follow-up, in contrast to the lowest scores observed in the pretest. Adolescents’ overall knowledge scores on all topics related to reproductive health (puberty, menstruation, diet, and exercise) differed significantly across the pretest, immediate posttest, and follow-up periods (F = 277.1, *p* = 0.0001).

Moreover, these findings align with those of Mahmoud & Ibrahim [22], who assessed the effectiveness of reproductive health education sessions for 63 blind and deaf students in Zagazig City. The study found that 100% of the participants had poor knowledge in the pretest, whereas 90.5% and 88.9% demonstrated good knowledge in the immediate posttest and follow-up, respectively. Additionally, there was a statistically significant difference between the mean scores of pretest, posttest, and follow-up knowledge (F = 850.3, *p* = 0.001).

The current study’s findings regarding the overall hygiene practices of the studied visually impaired adolescent females before, immediately after, and at follow-up of the educational sessions showed that, compared to over two-thirds of the SIM group, the vast majority of the Audio group had a healthy level of overall hygiene practices during menstruation at post-intervention and follow-up, with a highly statistically significant difference (*P* = 0.000).

The researchers suggest that hygiene practices improve as knowledge increases. Teenage girls who have sufficient understanding of bodily changes, menstrual hygiene management, healthy nutrition, and appropriate exercise are more capable of practicing all aspects related to reproductive health, which in turn enhances their overall well-being. However, the results of the current study indicate that audio educational sessions are a more effective method for improving health practices among visually impaired adolescent girls compared to the self-instructional module.

These results are consistent with research by Kumbhar & Bhore [19], which found that, during the pretest, 83.33% and 100% of blind adolescent girls had poor practice regarding reproductive health in the SIM group and Audio-CD ROM group, respectively, and that, during the posttest, 16.67% and 33.33% of SIM group and Audio-CD ROM group had average practice.

These results also align with the findings of a study conducted by El-Kurdy, Fadel and Elsayed [23], which found that the majority of visually challenged adolescent school-girls had poor practices regarding menstrual hygienic practice and improved after the structured audio-educational sessions, with highly statistically significant differences (*P* ≤ 0.00) between the two periods.

Moreover, these results match with the research conducted by Mohamed [24], who examined the “Effect of health education program on menstrual practices among secondary school females” and discovered that the majority of students demonstrated a healthy practice score level in relation to all menstrual hygiene aspects in the post-test compared to pre-test.

Furthermore, in the same direction, Ghazy & Fathy [20] demonstrated that, following an audio drama program, over 75% of visually impaired students had good health habits, with a statistically significant difference between the two study phases.

In addition, Bagirisano et al. [25] highlighted a significant improvement in menstrual hygiene knowledge and practices among visually impaired adolescent girls following the implementation of audio-recorded interventions. This indicates that such approaches can enhance knowledge, improve hygiene practices, and promote greater independence in self-care.

The present study’s findings indicate that, regarding the overall behaviors and limitations of the examined visually impaired adolescent females during menstruation, more than two-thirds of the Audio group demonstrated good overall behaviors at both post-intervention and follow-up audio educational sessions, whereas approximately two-thirds of the SIM group demonstrated good behaviors during post- and follow-up Braille educational sessions.

These results can be explained by the fact that improving general health practices among visually impaired adolescent girls contributed to enhancing their overall behaviors and managing limitations through the audio educational sessions.

These findings are corroborated by a study conducted by Faheim et al. [26], which reported that most female participants refrained from performing daily tasks such as household chores, attending school, exercising, and visiting places of worship during the pretest. Significant improvements were observed in these behaviors in the post-test following the implementation of audio educational sessions.

Additionally, these results are consistent with the study conducted by Jayanthi [6], which found that participants’ behaviors improved significantly after exposure to an audio drama intervention.

Furthermore, these findings align with the study by Deshpande et al. [27], which confirmed that the teaching strategies employed with blind adolescent girls resulted in measurable behavioral improvements between pre- and post-test I and II.

Thus, the present study’s results demonstrated that the hypothesis was supported, showing a significant increase in the levels of knowledge, hygienic practices, and behaviors regarding menstruation among visually impaired adolescent females in both groups following the implementation of audio educational sessions and the self-instructional module.

However, a key limitation of this study was the difficulty visually impaired adolescent girls faced in interpreting graphics, which may have affected their understanding and engagement. Although Braille materials, verbal explanations, and tactile resources were used to address this challenge, some participants may still have experienced limitations in fully accessing the information. Providing more detailed tactile diagrams, step-by-step verbal guidance, and individualized support can help enhance comprehension and engagement.

## Conclusion

Based on the present study findings, it can be concluded that the integration of a Braille-based self-learning module with audio educational sessions proved to be an effective strategy not only for improving menstrual knowledge but also for promoting healthier practices and positive behavioral changes among visually impaired adolescent girls.

### Study limitations

Difficulty in visualizing graphics and illustrations among visually impaired adolescent girls.

### Recommendation

Based on the present study findings, the following recommendations are suggested:

- To promote a culture of reproductive health among girls who will become mothers, the Ministry of Health and Population and the Ministry of Education should publish and distribute educational Braille booklets and recorded CDs for adolescent girls with visual impairments in all blind schools.
- Ongoing health education initiatives should be used to raise visually impaired adolescent girls’ knowledge of menstruation and associated issues using a self-education module with the Braille method and audio CD-ROM.
- Educational resources regarding menstruation and sanitary practices should be included in school libraries, along with brochures and an adequate number of Braille books.

### Further additional studies

Further research with larger samples and broader geographic coverage is needed to better understand the unique menstrual needs of visually impaired adolescent girls and girls with various impairments, taking cultural differences into account and including younger girls and their parents.

## Data Availability

Due to confidentiality concerns, the data and materials used in this study cannot be made publicly available. However, they can be obtained from the corresponding author upon reasonable request.

## Abbreviations

VI: Visually Impaired
SIM: Self-instructional Modules
WHO: World Health Organization
SES: Socioeconomic Status
